# Socio-demographic and -economic factors associated with 30-day readmission for conditions targeted by the hospital readmissions reduction program: a population-based study

**DOI:** 10.1186/s12889-021-11987-z

**Published:** 2021-10-23

**Authors:** Frances Murray, Meghan Allen, Collin M. Clark, Christopher J. Daly, David M. Jacobs

**Affiliations:** Department of Pharmacy Practice, School of Pharmacy and Pharmaceutical Sciences, University at Buffalo, Buffalo, NY USA

## Abstract

**Background:**

Early hospital readmissions remain common in patients with conditions targeted by the CMS Hospital Readmission Reduction Program (HRRP). There is still no consensus on whether readmission measures should be adjusted based on social factors, and there are few population studies within the U.S. examining how social characteristics influence readmissions for HRRP-targeted conditions. The objective of this study was to determine if specific socio-demographic and -economic factors are associated with 30-day readmissions in HRRP-targeted conditions: acute exacerbation of chronic obstructive pulmonary disease, pneumonia, acute myocardial infarction, and heart failure.

**Methods:**

The Nationwide Readmissions Database was used to identify patients admitted with HRRP-targeted conditions between January 1, 2010 and September 30, 2015. Stroke was included as a control condition because it is not included in the HRRP. Multivariate models were used to assess the relationship between three social and economic characteristics (gender, urban/rural hospital designation, and estimated median household income within the patient’s zip code) and 30-day readmission rates using a hierarchical two-level logistic model. Age-adjusted models were used to assess relationship differences between Medicare vs. non-Medicare populations.

**Results:**

There were 19,253,997 weighted index hospital admissions for all diagnoses and 3,613,488 30-day readmissions between 2010 and 2015. Patients in the lowest income quartile (≤$37,999) had an increased odds of 30-day readmission across all conditions (*P* < 0.0001). Female gender and rural hospital designation were associated with a decreased odds of 30-day readmission for most targeted conditions (*P* < 0.05). Similar findings were also seen in patients ≥65 years old.

**Conclusions:**

Socio-demographic and -economic factors are associated with 30-day readmission rates and should be incorporated into tools or interventions to improve discharge planning and mitigate against readmission.

## Introduction

Early hospital readmissions are associated with increased healthcare costs and remain common, particularly within the Medicare population [1, 2]. For example, one in five patients hospitalized with an acute exacerbation of chronic obstructive pulmonary disease (AECOPD) are rehospitalized within 30 days of discharge, of which 10–55% may be preventable [3–5]. High rates of preventable readmissions are not specific to AECOPD and also occur with other common comorbidities including heart failure (HF), acute myocardial infarction (AMI), and pneumonia (PNA) [2]. Given the potentially high number of preventable readmissions, the Centers for Medicare and Medicaid Services (CMS) implemented the Hospital Readmissions Reduction Program (HRRP) in 2012 [6]. This policy penalizes hospitals exceeding expected rates of readmission within 30 days of discharge. In 2013, 67% of hospitals were penalized due to excessive readmissions, a disproportionate number of which were safety net hospitals serving low-income patients [7]. The program was targeted to three conditions, AMI, HF, and PNA, and, in 2015, the policy was expanded to cover readmissions associated with AECOPD [6].

Social risk factors are important determinants of health outcomes and are disproportionately represented in high-needs populations [8, 9]; for example, the lowest socioeconomic groups are up to 14-times more likely to have respiratory disease [10]. Certain social characteristics such as race, ethnicity, socioeconomic status, place of residence, and disability may predict readmission risk, particularly in complex conditions such as HF and AMI [11–14]. Overall, low socioeconomic status and social disadvantage have been shown to be associated with an increased risk of readmission; however, there are few large population-level studies examining the relationships between sociodemographic and socioeconomic status and readmissions. From a policy perspective, controversy also exists as to whether readmissions measures used to reimburse hospitals should adjust for socioeconomic factors [15–21]. On the one hand, socioeconomic adjustment would avoid penalizing hospitals caring for disadvantaged patients, but on the other it could inadvertently excuse the delivery of substandard care to disadvantaged populations, committing hospitals to different standards for the outcomes of patients based on their socioeconomic background [17, 18, 20]. Alternatively, effective interventions based on a patient’s social determinants of health (SDoH) beyond the hospital will be necessary to ultimately prevent hospital readmissions [22]. Providers and hospitals recognize the association of social needs with patient outcomes, yet these groups may be reluctant to assume responsibility for a patient’s social-related needs given the complexity addressing these needs coupled with increasing clinical demands [23, 24]. Identifying and addressing a patient’s SDoH through the development of accessible and evidence-based programs will be needed as a complimentary approach to improve health outcomes.

Medical care only accounts for 10–20% of the modifiable contributors to health outcomes with up to 80% contributed through a patient’s SDoH [25–27]. Current readmission policy does not adequately account for patient sociodemographic factors, which may further drive health inequity [28]. An approach to address the current shortcomings may be a combination of policy changes and concurrently working towards implementation of community-level evidence-based interventions. Additional evidence is required showing differences in health care utilization based on socio-economic and -demographic characteristics to ultimately enrich risk adjustment models, advocate towards policy changes, or develop interventions so they reflect a patient’s whole health rather than just their comorbidity burden [29]. Therefore, the objective of this study was to determine whether three social and economic characteristics (gender, median income associated with a patient’s zip code, and urban/rural hospital designation) are associated with 30-day readmission in patients with HRRP-targeted conditions (AECOPD, AMI, HF, and PNA).

## Methods

### Data source

The Nationwide Readmissions Database (NRD) is one of the largest publicly-available all-payer databases in the United States. The NRD is derived from the Health Care Utilization Project’s (HCUP) State Inpatient Databases sponsored by the Agency for Healthcare Research and Quality (AHRQ). The NRD includes admission information for community hospitals but not rehabilitation and long-term acute care hospitals. The unweighted sample compiles approximately 50% of all U.S. hospitalizations. With the NRD’s complex survey design, sample weights are applied to raw data to generate national estimates. The University at Buffalo Institutional Review board exempted this study from review, as data were de-identified and publicly available through the AHRQ.

### Study sample

Data from the NRD compiled approximately 19 million weighted index admissions of individuals with a diagnosis of AECOPD, AMI, HF, and PNA. Stroke was studied as a control condition because it was not included in the HRRP. International Classification of Disease, Ninth Revision, Clinical Modification (ICD-9-CM) codes were used to identify patients with each of the five conditions between January 2010 and September 2015. After September 2015, International Classification of Disease, Tenth Revision (ICD-10) codes were implemented, so these data were excluded to avoid code inconsistencies. Additionally, we limited our index hospitalization to January 1 to November 30, 2010–2014 in order to capture all 30 day readmissions. ICD-9-CM codes were selected based on the codes created for the CMS for the HRRP to assess all-cause readmissions for AECOPD, HF, AMI, PNA, and stroke [30–33]. Adults 18 years and older with the diagnoses of HF, AMI, PNA, and stroke were included. Only adults ≥40 years of age with a diagnosis of AECOPD were identified for our study sample to be consistent with previous COPD readmission studies [34–36]. Patients were excluded if they died during the index hospitalization, were discharged against medical advice, whose length of stay was missing, whose readmission was elective, or who were discharged to another acute care facility.

### Primary exposures

The primary exposures were three socio-demographic and -economic factors: (i) patient gender (male vs. female); (ii) hospital’s urban/rural designation; and (iii) median income associated with patient zip code. Urban/rural designation was based on the county of the hospital as identified by the American Hospital Association and categorized as urban (large and small metropolitan) or rural (micropolitan and not metropolitan) [37]. This categorization is consistent with the Urban Influence Codes utilized by the NRD, which groups large and small metropolitan areas as “metropolitan” and micropolitan and non-metropolitan areas as “not metropolitan”. The NRD categorizes income into quartiles based on the estimated median household income of residents in the patient’s zip code ($1–37,999, $38,000–$47,999, $48,000–$63,999, and ≥ $64,000) derived from zip code-demographic data obtained from Claritas [37]. The estimated median household income data were reported in 2013 dollars.

### Covariates

Covariates were included due to their potential to impact 30-day readmissions and were stratified based on subgroup. Patient and hospital characteristics included age, comorbidities, insurance type, and index length of stay. Age was stratified into 18–39, 40–64, 65–74, and 75–90 years. After reviewing the current literature, specific comorbidities for these populations were analyzed such as congestive heart failure, diabetes (uncomplicated), hypertension (uncomplicated and complicated), and renal failure. Insurance type was classified as Medicare, Medicaid, private, and self-pay/no charge. The Elixhauser Comorbidity Index was utilized to develop mortality and readmission scores based on the 29 comorbidity variables in the HCUP database [38]. Current NRD comorbidities were altered to match the Elixhauser Comorbidity Index scores utilized by the HCUP.

### Outcomes

The primary outcome was unplanned all-cause 30-day readmission, consistent with definitions set by the CMS HRRP measures [39]. The 30-day readmission variable was defined as any patient with a readmission 1–30 days after discharge from an index hospitalization. These readmissions were not counted as a new index hospitalization. All additional hospitalizations during the 30-day period were not included as a 30-day readmission for the same condition. Hospitalizations occurring after this 30-day period were considered an index hospitalization. This consistent with HRRP policy for counting readmissions.

### Statistical analysis

To account for the complex survey design of the NRD, we used a survey-specific methodology with hospital as clusters, NRD stratum as strata, and discharge-level weights as weights to obtain study population data and weighted nationwide overall and annual 30-day readmission rates for each targeted condition. Baseline demographic and hospital characteristics were summarized into two groups based on whether the subject was readmitted within 30 days. Categorical variables were reported as percentages within each targeted condition. Hierarchical two-level logistic models with hospital ID as a random effect were used to evaluate the relationships between the targeted conditions and 30-day readmission. All models were adjusted for demographic variables, hospital characteristics, and Elixhauser comorbidity and mortality risk scores. Five separate multivariable models were run for each condition. An age-adjusted model was created for the social characteristics with the < 65 and ≥ 65 year age groups to analyze readmission rates in both the Medicare population (the policy target group) and the non-Medicare population. All statistical tests were two-tailed with a *P* < 0.05 level of significance and analyses were conducted using SAS version 9.4 (SAS Institute, Cary, NC).

## Results

### Sample demographics

There were 19,253,997 weighted index hospital admissions for all diagnoses and 3,613,488 30-day readmissions between 2010 and 2015. Table 1 presents demographic data stratified by condition and readmission event. Readmissions were approximately equal for males and females across conditions except for AECOPD, where women accounted for 56% of readmissions, and for AMI, where men accounted for 56% of readmissions. Hospital designation was consistent across all five conditions, with approximately 80–90% of patients visiting hospitals in urban areas. Estimated median household income was similar across conditions, with approximately one third of patients falling into the lowest income category (≤$37,999). Patients aged 75 to 90 years accounted for the highest percentage of readmissions except for AECOPD, where patients aged 40 to 64 years had the highest readmission rates.

**Table 1:** Baseline clinical and demographic characteristics of index hospitalizations for patients with chronic obstructive pulmonary disease, pneumonia, acute myocardial infarction, and stroke^a^

	COPD (*N* = 4,198,163)		PNA (*N* = 6,312,433)		AMI (*N* = 2,384,654)		HF (N = 4,496,384)		Stroke (*N* = 1,862,363)	
	Readmit (%) (***N*** = 893,376)	Non-readmit (%) (***N*** = 3,304,787)	Readmit (%) (***N*** = 1,045,993)	Non-readmit (%) (***N*** = 5,266,440)	Readmit (%) (***N*** = 355,040)	Non-readmit (%) (***N*** = 2,029,614)	Readmit (%) (***N*** = 1,080,492)	Non-readmit (%) (***N*** = 3,415,891)	Readmit (%) (***N*** = 238,587)	Non-readmit (%) (***N*** = 1,623,776)
**Gender**										
Female	56	59	49	51	44	37	49	50	51	52
Male	44	41	51	49	56	63	51	50	49	48
**Hospital Urban/Rural Designation**										
Urban	83	81	84	82	91	92	87	86	90	89
Rural	17	19	16	18	9	8	13	14	10	11
**Estimated Median Household Income by Zip Code**										
≥ $64,000	14	14	19	19	18	19	17	18	19	20
$48,000–$63,999	20	21	23	23	22	23	21	22	23	23
$38,000–$47,999	26	27	25	26	26	26	25	25	25	25
≤ $37,999	38	36	32	31	32	30	36	33	32	31
**Age**										
18–39	N/A	N/A	5	6	2	3	3	2	2	2
40–64	39	39	26	26	32	44	27	25	28	32
65–74	29	29	21	18	24	23	22	20	23	22
75–90	32	32	45	42	43	31	49	52	47	44
**Comorbidities**										
CHF	35	26	30	21	–	–	–	–	18	12
Diabetes	31	27	26	22	33	29	25	34	32	29
Hypertension	69	67	62	56	75	72	70	70	83	82
Renal Failure	17	12	24	16	31	17	50	41	20	13
**Insurance Type**										
Medicare	73	70	74	64	70	55	77	76	72	66
Medicaid	14	12	12	13	8	7	11	8	9	8
Private	7.9	12	11	16	15	28	8	11	14	18
Self-pay/No Charge/Other	4.4	6	4	6	6	11	4	6	6	8
**Length of Stay (days)**										
≤ 2	24	29	14	23	24	38	22	26	23	33
3–5	44	46	39	44	39	39	42	44	40	42
> 5	32	25	47	32	36	22	36	29	36	23
**Number of Comorbidities**										
≤ 5	27	36	35	46	18	32	13	17	24	33
6–7	26	27	25	22	25	29	23	26	27	29
8–9	22	19	20	15	24	20	26	26	23	20
> 9	25	18	20	12	33	19	38	32	26	18

*Abbreviations*: *COPD* chronic obstructive pulmonary disease, *PNA* pneumonia, *AMI* acute myocardial infarction, *HF* heart failure, *CHF* congestive heart failure

^a^ Readmit was defined as those patients that required a hospital readmission within 30 days of the index admission. All other patients were included in the non-readmit category

### Chronic obstructive pulmonary disease

**Fig. 1:**
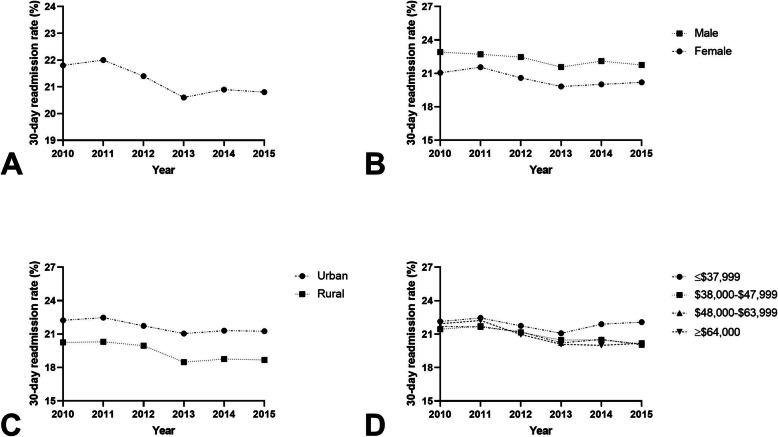
**A** 30-day readmission rates as a percentage of total hospitalizations for AECOPD; **B** 30-day readmission rates for males and females as a percentage of total hospitalizations for AECOPD (*P* < 0.001); **C** 30-day readmission rates for rural and urban hospitals as a percentage of total hospitalizations for AECOPD (*P* < 0.001); **D** 30-day readmission rates for estimated median household income ≤$37,999, $38,000–$47,999, $48,000–$63,999, and ≥ $64,000 as a percentage of total hospitalizations for AECOPD (*P* < 0.001)

For AECOPD, there were 4,198,163 index hospital admissions and 893,376 (21.3%) 30-day readmissions between 2010 and 2015 (Fig. 1A). During the period, the readmission rate among females was 20.6% (range, 19.8–21.6%) compared to 22.2% (range, 21.6–22.9%) [*p* < 0.001; Fig. 1B]. In adjusted models, female gender was associated with an 11% reduced odds of 30-day readmission (aOR 0.889; 95% CI 0.885–0.893, *P* < 0.0001) (Table 2). Readmissions among urban hospitals was 21.7% (range, 21.0–22.2%) compared to 19.5% (range, 18.7–20.3%) in rural hospitals (*p* < 0.001; Fig. 1C). Patients admitted to rural hospitals had a 8% reduced odds of 30-day readmission compared to those admitted to urban hospitals (aOR 0.916; 95% CI 0.910–0.923 *P* < 0.0001). An estimated median household income ≤$37,999 had a readmission rate of 21.9% (range, 21.0–22.5%) and these individuals had a 1.05-times increased odds of 30-day readmission compared to the highest median household income quartile (aOR 1.053; 95% CI 1.045–1.062, *P* < 0.0001; Fig. 1D).

**Fig. 2:**
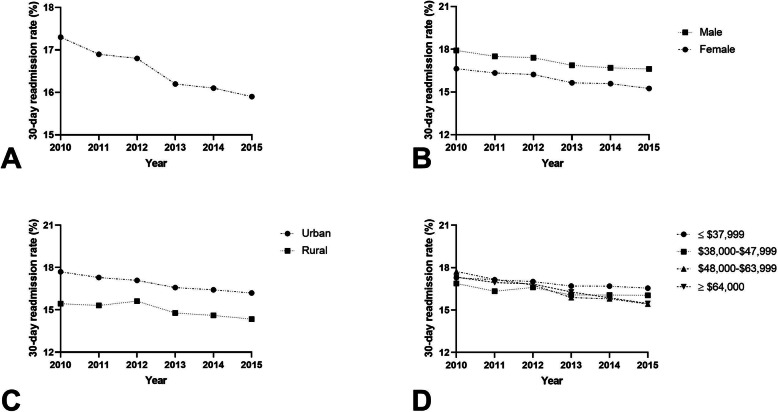
**A** 30-day readmission rates as a percentage of total hospitalizations for pneumonia; **B** 30-day readmission rates for males and females as a percentage of total hospitalizations for pneumonia(*P* < 0.001); **C** 30-day readmission rates for rural and urban hospitals as a percentage of total hospitalizations for pneumonia (*P* < 0.001); **D** 30-day readmission rates for estimated median household income ≤$37,999, $38,000–$47,999, $48,000–$63,999, and ≥ $64,000 as a percentage of total hospitalizations for pneumonia (*P* < 0.001)

**Table 2:** Multivariable analysis of associations between 30-day readmission and social risk factors in chronic obstructive pulmonary disease, pneumonia, acute myocardial infarction, heart failure, and stroke^a,b,c^

Characteristic	AECOPD		PNA		AMI		HF		Stroke	
	aOR (95% CI)	***P***	aOR (95% CI)	***P***	aOR (95% CI)	***P***	aOR (95% CI)	***P***	aOR (95% CI)	***P***
**Gender**										
Male	1.0 (Reference)		1.0 (Reference)		1.0 (Reference)		1.0 (Reference)		1.0 (Reference)	
Female	0.889 (0.885–0.893)	<0.0001	0.906 (0.903–0.910)	<0.0001	1.115 (1.106–1.123)	<0.0001	0.978 (0.973–0.982)	<0.0001	0.935 (0.927–0.944)	<0.0001
**Hospital Urban-Rural Designation**										
Urban	1.0 (Reference)		1.0 (Reference)		1.0 (Reference)		1.0 (Reference)		1.0 (Reference)	
Rural	0.916 (0.910–0.923)	<0.0001	0.915 (0.909–0.920)	<0.0001	1.028 (1.015–1.042)	<0.0001	0.972 (0.965–0.979)	<0.0001	0.944 (0.930–0.958)	<0.0001
**Estimated Median Household Income by Zip Code**										
≥ $64,000	1.0 (Reference)		1.0 (Reference)		1.0 (Reference)		1.0 (Reference)		1.0 (Reference)	
$48,000–$63,999	0.996 (0.987–1.004)	0.2847	1.013 (1.007–1.020)	<0.0001	1.001 (0.989–1.013)	0.887	1.018 (1.011–1.026)	<0.0001	1.005 (0.991–1.019)	0.4715
$38,000–$47,999	1.009 (1.001–1.017)	0.213	1.020 (1.013–1.027)	<0.0001	1.027 (1.016–1.039)	<0.0001	1.032 (1.025–1.039)	<0.0001	1.002 (0.989–1.016)	0.7590
≤ $37,999	1.053 (1.045–1.062)	<0.0001	1.066 (1.059–1.073)	<0.0001	1.063 (1.051–1.075)	<0.0001	1.104 (1.096–1.111)	<0.0001	1.045 (1.031–1.059)	<0.0001
**C-statistic**	0.602 (0.601, 0.603)	<0.0001	0.634 (0.633, 0.635)	<0.0001	0.667 (0.665, 0.668)	<0.0001	0.588 (0.587, 0.589)	<0.0001	0.623 (0.621, 0.625)	<0.0001

^a^ Each estimate is adjusted for all other variables in the model

^b^ Additional covariates in each model: Elixhauser comorbidities score (n = 29); Elixhauser mortality score (*n* = 29); length of stay (categorical); age (categorical), expected primary payer, number of chronic conditions

^c^ Survey weights applied to give national estimates

*Abbreviations*: *aOR* adjusted odds ratio, *AECOPD* acute exacerbation of chronic obstructive pulmonary disease, *PNA* pneumonia, *AMI* acute myocardial infarction, *HF* heart failure

### Pneumonia

There were 6,312,433 index hospital admissions and 1,045,993 (16.6%) 30-day readmissions for PNA (Fig. 2A). Females had a readmission rate of 15.9% (range, 15.3–16.6%) compared to 17.2% (range, 16.6–17.9%) among males (*p* < 0.001; Fig. 2B). In adjusted models, females had a 9% reduced odds of 30-day readmission compared to males (aOR 0.906; 95% CI 0.903–0.910, *P* < 0.0001) (Table 2). The readmission rate among patients admitted to rural hospitals was 15.1% (range, 14.3–15.4%) compared to 16.9% (range, 16.2–17.7%) among urban hospitals (p < 0.001; Fig. 2C). Patients treated at rural hospitals had a 8% reduced odds of 30-day readmission compared to those admitted to urban hospitals (aOR 0.915; 95% CI 0.909–0.920, *P* < 0.0001). There was a significant trend towards higher readmission rates based on income quartile (Fig. 2D). Median household income was associated with an increased odds of readmission for income quartile ≤$37,999 (aOR 1.066; 95% CI 1.059–1.073, *p* < 0.0001) and $38,000–$47,999 (aOR 1.020; 95% CI 1.013–1.027, *P* < 0.0001).

### Acute myocardial infarction

**Fig. 3:**
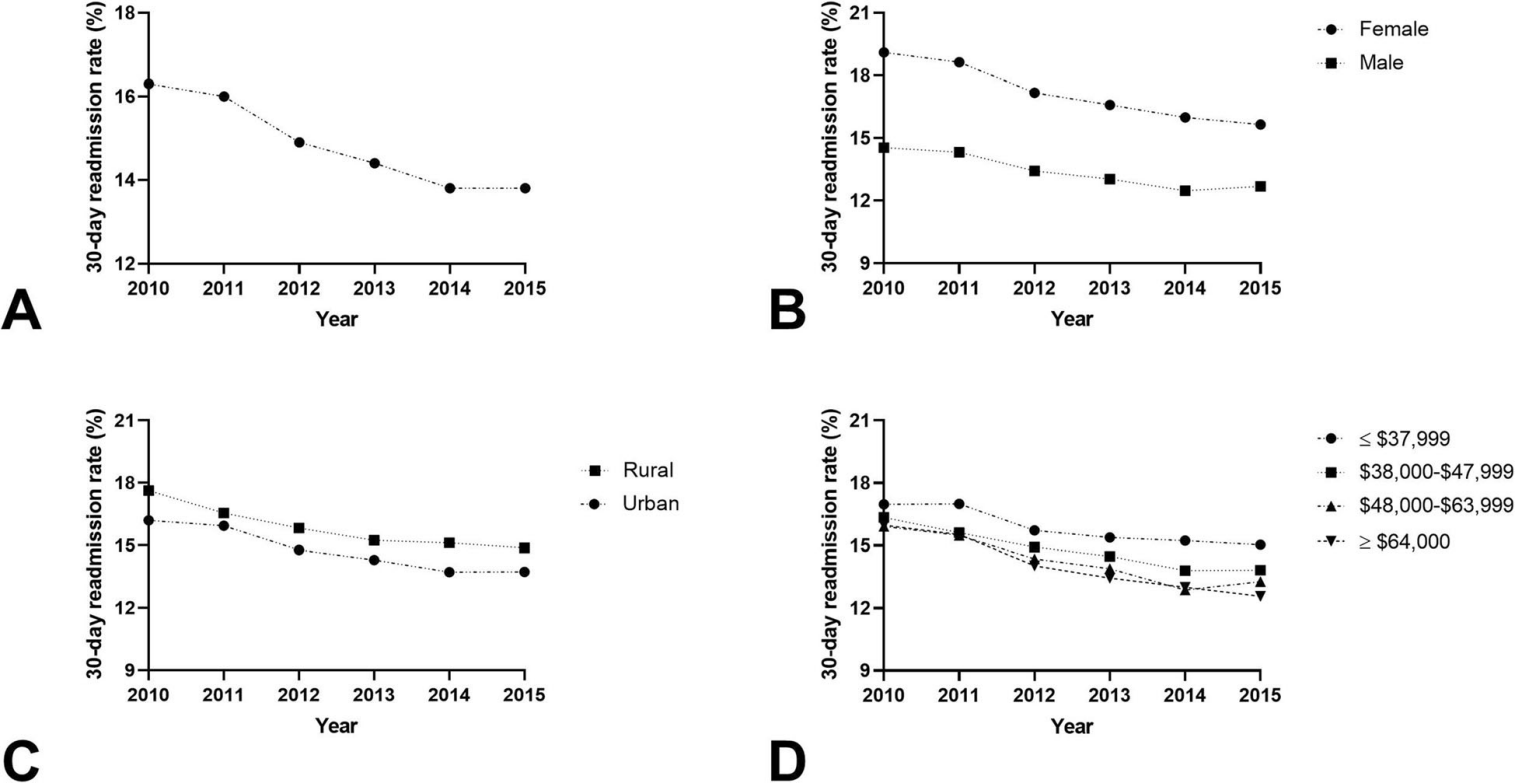
**A** 30-day readmission rates as a percentage of total hospitalizations for AMI; **(B)** 30-day readmission rates for males and females as a percentage of total hospitalizations for AMI (*P* < 0.001); **C** 30-day readmission rates for rural and urban hospitals as a percentage of total hospitalizations for AMI (*P* < 0.001); **D** 30-day readmission rates for estimated median household income ≤$37,999, $38,000–$47,999, $48,000–$63,999, and ≥ $64,000 as a percentage of total hospitalizations for AMI (*P* < 0.001)

There were 2,384,654 index hospital admissions and 355,040 (14.9%) 30-day readmissions for patients diagnosed with AMI (Fig. 3A). Females had a readmission rate of 17.2% (range, 15.6–19.1%) compared to 13.4% (range, 12.7–14.5%) among males (*P* < 0.001; Fig. 3B). Adjusted models showed that females had a 1.115-times increased odds of early readmission than males (aOR 1.115; 95% CI 1.106–1.123, *P* < 0.0001) (Table 2). Patients treated at urban hospitals had a readmission rate of 14.8% (range, 13.7–16.2%) compared to a 15.9% (range, 14.9% - 17.6) readmission rate at rural hospitals (*P* < 0.001; Fig. 3C). There was a 1.028-times increased odds of 30-day readmission for patients admitted to rural hospitals compared to those admitted to urban hospitals (aOR 1.028; 95% CI 1.015–1.042, *P* < 0.0001). Those in the lowest income quartile had an elevated readmission rate (15.9%, range, 15.05–16.9%) compared to patients in in the highest quartile (14.1%, range, 12.6–16.0%, *P* < 0.001; Fig. 3D). Patients with an estimated median household income of ≤$37,999 had a 1.063-times increased odds of 30-day readmission (aOR 1.063; 95% CI 1.051–1.075, *P* < 0.0001).

### Heart failure

**Fig. 4:**
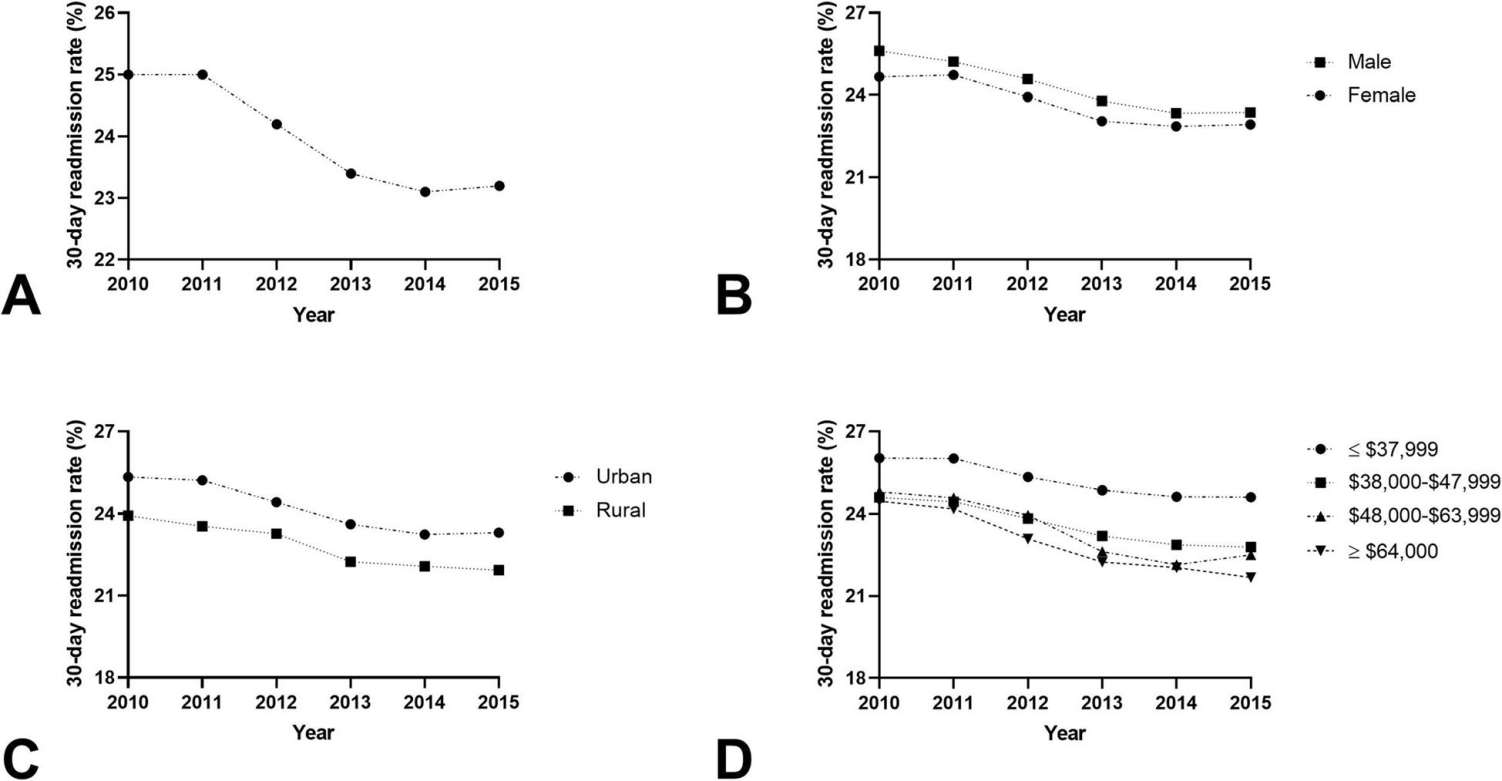
**A** 30-day readmission rates as a percentage of total hospitalizations for heart failure; **B** 30-day readmission rates for males and females as a percentage of total hospitalizations for heart failure (*P* < 0.001); **C** 30-day readmission rates for rural and urban hospitals as a percentage of total hospitalizations for heart failure (*P* < 0.001); **D** 30-day readmission rates for estimated median household income ≤$37,999, $38,000–$47,999, $48,000–$63,999, and ≥ $64,000 as a percentage of total hospitalizations for heart failure (*P* < 0.001)

There were 4,496,384 index hospitalizations and 1,080,492 (24%) 30-day readmissions for HF, the highest 30-day readmission percentage of all five targeted conditions analyzed (Fig. 4A**).** Females had a readmission rate of 23.7% (range, 22.9–24.7%) compared to 24.3% (range, 23.3–25.6%) among males (*p* < 0.001; Fig. 4B). In adjusted models, females had a 2% reduced odds of 30-day readmission compared to males (aOR 0.978; 95% CI 0.973–0.982, *P* < 0.0001) (Table 2). Patients seen in rural areas had a lower readmission rate (22.9%, range, 21.9–23.9%) compared to urban areas (24.2%, range, 23.2–25.3%, *p* < 0.001, Fig. 4C). All median household income groups showed a significantly increased odds of 30-day readmission compared to those in the highest income quartile (*P* < 0.001) (Fig. 4D).

### Stroke

**Fig. 5:**
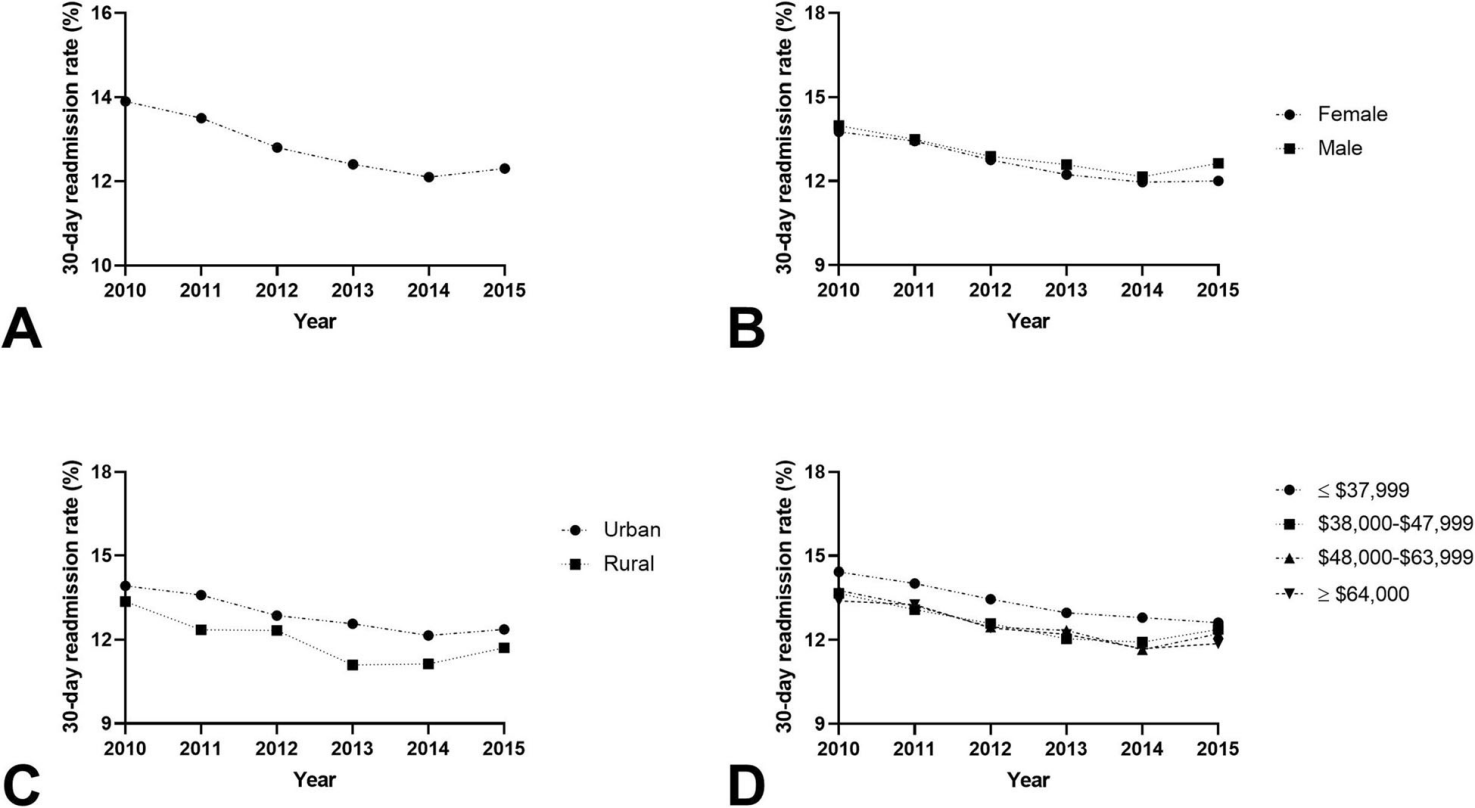
**A** 30-day readmission rates as a percentage of total hospitalizations for stroke; **B** 30-day readmission rates for males and females as a percentage of total hospitalizations for stroke (p < 0.001); **C** 30-day readmission rates for rural and urban hospitals as a percentage of total hospitalizations for stroke (p < 0.001); **D** 30-day readmission rates for estimated median household income ≤$37,999, $38,000–$47,999, $48,000–$63,999, and ≥ $64,000 as a percentage of total hospitalizations for stroke (*p* < 0.001)

There were 1,862,363 index hospitalizations for stroke and 238,587 (12.8%) 30-day readmissions (Fig. 5A**).** Female gender had a readmission rate of 12.7% (range, 12–13.8%) compared to 12.9% (range, 12.2–14%) among male gender (P < 0.001; Fig. 5B). In adjusted models, females had a 6% reduced odds of 30-day readmission compared to men (aOR 0.935; 95% CI 0.927–0.445, *P* < 0.0001) (Table 2). Patients seen at rural hospitals had a readmission rate of 12% (range, 11.1–13.4%) compared to 12.9% (range, 12.2–13.9%) at urban hospitals (p < 0.001; Fig. 5C). Rural hospitals had a 5% reduced odds of early readmission compared to urban hospitals (aOR 0.944; 95% CI 0.930–0.958, *P* < 0.0001) (Table 2). The readmission rate was 13.4% (range, 12.6–14.4%) in patients with an estimated median household income ≤$37,999, who had a significantly increased odds of 30-day readmission compared to the highest income quartile (aOR 1.045; 95% CI 1.031–1.059, *P* < 0.0001).

### Age-adjusted analyses between Medicare and non-Medicare populations

Males with a diagnosis of COPD and < 65 years old had an increased odds of early readmission (aOR 1.17; 95% CI 1.16–1.18, *P* < 0.0001) compared to the other four target conditions (Table 3). Hospitals with urban designations, a diagnosis of COPD, and < 65 years old were associated with an increased odds of early readmission (aOR 1.13; 95% CI 1.11–1.15, *P* < 0.0001). With respect to median household income, those with the greatest odds of early readmission were patients diagnosed with HF aged ≥65 years and with ≤$37,999 total income. The adjusted odds ratios between the defined age groups were also notable: females diagnosed with a stroke and < 65 years old had an increased odds of readmission (aOR 1.042; 95% CI 1.017–1.068, *p* < 0.0001) compared to those ≥65 years old (aOR .899; 95% CI 0.885–0.913, *P* < 0.0001). Patients diagnosed with pneumonia and < 65 years old also had a decreased odds of readmission in all median household income categories, and those ≥65 years old all had an increased odds of early readmission.

**Table 3:** Age-adjusted associations between 30-day readmission and social risk factors in chronic obstructive pulmonary disease, pneumonia, acute myocardial infarction, heart failure, and stroke ^a,b^

	AECOPD		HF		AMI		PNA		Stroke	
	< 65 years	≥65 years	< 65 years	≥65 years	< 65 years	≥65 years	< 65 years	≥65 years	< 65 years	≥65 years
**Gender**										
Female	0.86 (0.85–0.87)*	0.92 (0.91–0.93)*	0.965 (0.95–0.98)*	0.98 (0.97–0.99)*	1.16 (1.14–1.18)*	1.10 (1.09–1.12)*	0.91 (0.90–0.92)*	0.91 (0.90–0.91)*	1.04 (1.02–1.07)*	0.90 (0.89–0.91)*
Male	1.17 (1.16–1.18)*	1.09 (1.08–1.10)*	1.037 (1.02–1.05)*	1.02 (1.02–1.03)*	0.86 (0.84–0.0.88)*	0.91 (0.90–0.92)*	1.10 (1.09–1.11)*	1.10 (1.10–1.11)*	0.96 (0.94–0.98)*	1.11 (1.10–1.13)*
**Hospital Urban/Rural Designation**										
Urban	1.13 (1.11–1.15)*	1.04 (1.02–1.05)*	1.041 (1.02–1.07)*	0.99 (0.98–1.01)	0.97 (0.93–1.01)	0.95 (0.92–0.97)*	1.08 (1.06–1.10)*	1.03 (1.02–1.04)*	1.04 (0.99–1.09)	1.06 (1.03–1.09)*
Rural	0.88 (0.87–0.90)*	0.97 (0.95–0.98)*	0.961 (0.94–0.98)*	1.01 (0.99–1.02)	1.04 (0.99–1.08)	1.06 (1.03–1.09)*	0.92 (0.91–0.94)*	0.97 (0.96–0.98)*	0.96 (0.92–1.01)	0.95 (0.92–0.97)*
**Median Household Income**										
≥ $64,000	1.0	(Reference)	1.0	(Reference)	1.0	(Reference)	1.0	(Reference)	1.0	(Reference)
$48,000–$63,999	0.99 (0.96–1.01)	1.00 (0.98–1.01)	1.018 (0.99–1.04)	1.02 (1.01–1.03)*	1.01 (0.98–1.04)	1.01 (0.99–1.03)	0.96 (0.95–0.98)*	1.01 (1.00–1.03)*	1.02 (0.98–1.06)	1.00 (0.98–1.02)
$38,000–$47,999	0.98 (0.96–1.00)	1.01 (1.00–1.03)	1.030 (1.01–1.05)*	1.05 (1.04–1.06)*	1.03 (1.00–1.07)*	1.04 (1.02–1.06)*	0.96 (0.94–0.98)*	1.04 (1.02–1.05)*	0.98 (0.95–1.02)	1.01 (0.99–1.03)
≤ $37,999	1.01 (0.99–1.03)	1.06 (1.05–1.08)*	1.085 (1.06–1.11)*	1.12 (1.11–1.14)*	1.07 (1.04–1.10)*	1.07 (1.05–1.09)*	0.96 (0.95–0.98)*	1.09 (1.08–1.10)*	0.95 (0.92–0.99)*	1.06 (1.04–1.09)*

* Indicates statistical significance at *P* < 0.05

^a^ Each estimate is adjusted for all other variables in the model

^b^ Additional covariates in each model: Elixhauser comorbidities score (*n* = 29); Elixhauser mortality score (n = 29); length of stay (categorical); age (categorical), expected primary payer, number of chronic conditions

*Abbreviations*: *AECOPD* acute exacerbation of chronic obstructive pulmonary disease, *HF* heart failure, *AMI* acute myocardial infarction, *PNA* pneumonia

## Discussion

This study utilized the National Readmissions Database to evaluate the impact of social characteristics on 30-day readmission rates for targeted conditions. For the targeted conditions, lowest income quartile, male gender, and urban hospital designation were associated with an increased odds of 30-day readmission. Within the age-adjusted analysis, there were significant differences in the risk factors for early readmission for different targeted conditions. Social and economic disparities will continue to play an important role in patient care and health outcomes. Current evidence suggests that up to 80% of a patient’s health outcomes are a result of social, behavioral, and economic factors rather than their medical care [25–27]. We have identified social and economic factors that continue to support the potential differences in health outcomes based on these social determinants of health.

A major criticism of the HRRP is that hospitals in low socioeconomic areas incur more penalties for treating more complex patients. This is especially problematic for safety net hospitals. These hospitals serve medically and socially vulnerable patients and consequently have higher 30-day readmission rates [40]; safety net hospitals could therefore be disproportionately fined by the HRRP due to the populations they serve [41]. Maddox and colleagues argued that social risk is outside a hospital’s control and hospitals should therefore not be penalized for treating patients at higher risk [42]. Social factors may be associated with higher readmission rates due to post-discharge healthcare access issues. Individuals with disproportionate social disadvantages may be unable to afford prescriptions, lack adequate transportation for follow-up appointments [43–45], have poor health literacy [45, 46], or be unable to follow self-care regimens [45, 47]. Attention to the association of social needs with medical outcomes is widespread, but dissemination of care delivery innovations in hospitals and medical practice is deficient [23, 48, 49]. Focusing post-discharge interventions on patient-related social needs will be just as important as any policy adjustment related to the CMS hospital readmission program [22]. This increasing recognition of the importance of social and health inequity justifies the development for accessible program that incorporate whole-person healthcare.

Patients in the lowest income quartile were more likely to be readmitted, consistent with previous literature [50–52]. Low socioeconomic status has been associated with low health literacy, poor social support, and a higher prevalence of comorbidities such as hypertension, diabetes, and obesity [53]. The higher prevalence of these conditions contributes to comorbid burden and results in complex patients who may be more difficult to treat. Low income has also been linked to disparities in healthcare access; patients with low incomes may have trouble affording medications and obtaining transportation to appointments [53], factors independent of the quality of care received at a hospital. Knowing that low income patients are at risk of early readmission could become an important consideration in transition of care programs. Socially vulnerable patients enrolled in community support-based transition of care programs have been shown to be less likely to use acute care and experience multiple readmissions and more likely to receive timely post-acute care and received higher quality discharge information [45, 54].

Female gender was associated with lower 30-day readmission rates for most targeted conditions, which may be due to greater utilization of preventative healthcare and healthcare in general. Females use significantly more preventative care including blood pressure checks, influenza immunizations, and cholesterol checks [53, 55]. Women are thought to establish better routine care and be more active in their own healthcare since they are exposed to routine healthcare at a younger age than men, receive prenatal care, and are screened more often than men [56]. Women are also more likely to be responsible for their children’s healthcare [55]. Despite receiving better care overall, the quality of care received by women in acute settings may be sub-optimal as compared to men [57]. Therefore, even though women have lower 30-day readmission rates, this is not the result of quality care in the acute setting but rather the result of better healthcare practices in the outpatient setting. Hospitals that underperform in providing care to women would therefore not be penalized under the HRRP. Similarly, hospitals that provide high-quality acute care to men could potentially be penalized if those male patients do not pursue post-acute care. Since post-acute care is heavily linked to income and social support, socially vulnerable patients are more likely to forgo post-acute care and are more likely to be readmitted.

Patients admitted to rural hospitals were less likely to be readmitted within 30 days of discharge from an acute care facility. There are conflicting findings on the relationship between hospital location and readmission rates. The targeted conditions studied here typically require follow-up with a specialist, and these specialists are often located only in urban centers [58]. Further, these early physician follow-ups are associated with lower 30-day readmission rates [59], so urban residents are likely to have better access to post-discharge specialized care and would therefore be less likely to be readmitted. Urban hospitals also tend to be larger and have additional infrastructure and established clinical protocols, suggesting that the quality of acute care may be better in these hospitals [60]. Conversely, it has also been reported that rural patients requiring additional resources or needing surgery are more likely to be treated at urban hospitals [61]. Therefore, clinical variables that measure the need for specialized care can serve as predictors of patient crossover from rural to urban hospitals. Urban hospitals may be associated with a higher odds of 30-day readmission because these hospitals see complex patients from both rural and urban areas who require more resources.

The findings of our study should be interpreted in the context of important limitations. First, we relied on CMS algorithms using ICD-9-CM codes to classify hospitalizations for our targeted conditions. The selections for the ICD-9-CM codes-based algorithms may have led to an underestimation of the number of hospitalizations for the targeted conditions. Since this methodology is used by the CMS to identify hospital admissions, we felt it was prudent to apply it to the entire study to provide national readmission estimates across age groups. Due to a limitation in the NRD, we excluded patients who were residents of different states. Persons are identified and tracked in the NRD with state-specific linkage numbers; therefore, a person readmitted between two different states cannot be tracked between states. This study was also limited by the inability to fully characterize social-related factors that may affect readmission such as access to transportation, social support, race and ethnicity, and health literacy. Extensive information on social risk factors are typically not collected as discrete data within inpatient databases, therefore limiting our analysis to available NRD data elements. Further research is needed to evaluate the confluence of social barriers and its impact on hospital readmission.

## Conclusion

In conclusion, our population study of HRRP-targeted conditions indicates differences in readmission rates based on certain social and economic conditions. Exploring policy changes, innovative care delivery models, and tools that address social and economic needs at hospital discharge should be explored and tested to mitigate against hospital readmissions. Ultimately, a complimentary approach between policy and implementing accessible, evidence based SDoH programs will be necessary to improve patient care and minimize health inequity.

## Data Availability

The data analyzed during the current study are available in the Nationwide Readmissions Database - https://www.hcup-us.ahrq.gov/nrdoverview.jsp
